# German tariffs for the ICECAP-Supportive Care Measure (ICECAP-SCM) for use in economic evaluations at the end of life

**DOI:** 10.1007/s10198-020-01260-2

**Published:** 2021-01-21

**Authors:** Judith Dams, Elisabeth Huynh, Steffi Riedel-Heller, Margrit Löbner, Christian Brettschneider, Hans-Helmut König

**Affiliations:** 1 Department of Health Economics and Health Services Research, Hamburg Center for Health Economics, University Medical Center Hamburg-Eppendorf, Martinistr. 52, 20246 Hamburg, Germany; 2 Department of Health Service Research and Policy, Research School of Population Health, Australian National University, Canberra, Australia; 3 Institute of Social Medicine, Occupational Health and Public Health (ISAP), University of Leipzig, Leipzig, Germany

**Keywords:** Value set, Capability, ICECAP-SCM, End-of-life, Best–worst scaling, Discrete choice experiment

## Abstract

**Objectives:**

Economic evaluations often use preference-based value sets (tariffs) for health-related quality of life to quantify health effects. For wellbeing at the end of life, issues beyond health-related quality of life may be important. Therefore, the ICECAP Supportive Care Measure (ICECAP-SCM), based on the capability approach, was developed. A validated German ICECAP-SCM version was published recently. However, tariffs for the German ICECAP-SCM are not available. Therefore, the aim was to determine tariffs for the ICECAP-SCM based on preferences of the German general population.

**Methods:**

An online sample of 2996 participants completed a best–worst scaling (BWS) and a discrete choice experiment (DCE). BWSs required participants to choose the best and worst statement within the same capability state, whereas DCEs required participants to trade-off between two capability states. First, BWS and DCE data were analyzed separately. Subsequently, combined data were analyzed using scale-adjusted conditional logit latent class models. Models were selected based on the stability of solutions and the Bayesian information criterion.

**Results:**

The two latent class model was identified to be optimal for the BWS, DCE, and combined data, and was used to derive tariffs for the ICECAP-SCM capability states. BWS data captured differences in ICECAP-SCM scale levels, whereas DCE data additionally explained interactions between the seven ICECAP-SCM attributes.

**Discussion:**

The German ICECAP-SCM tariffs can be used in addition to health-related quality of life to quantify effectiveness in economic evaluations. The tariffs based on BWS data were similar for Germany and the UK, whereas the tariffs based on combined data varied. We would recommend to use tariffs based on combined data in German evaluations. However, only results on BWS data are comparable between Germany and the UK, so that tariffs based on BWS data should be used when comparing results between Germany and the UK.

**Supplementary Information:**

The online version contains supplementary material available at 10.1007/s10198-020-01260-2.

## Introduction

According to current guidelines for clinical trials and economic evaluations, patient-reported outcomes should be evaluated in addition to clinical outcomes [1]. Most patient-reported outcomes focus on health-related quality of life. However, studies focusing on health outcomes like health-related quality of life neglect other relevant issues of wellbeing, such as personal wishes or needs [2, 3]. In particular, the exclusive focus on aspect of health-related quality of life becomes less relevant for informing decision-making at the end of life. Instead, more care is needed and persons often suffer physically and have individual needs, such as financial issues, as well as wishes for the funeral or wellbeing of family and friends [4–6]. Therefore, measures of health-related quality of life may be less suitable to assess effects of interventions at the end of life [7]. Consequently, other concepts are important to measure effects in economic evaluations of interventions at the end of life, especially for groups of people whose needs are insufficiently reflected by the concept of health-related quality of life.

In contrast to the concept of health-related quality of life, the capability approach offers the possibility to focuses on the capability of persons to achieve wellbeing instead of utility [8–10]. Thus, the core focus is on what persons are able to do and who they are able to be, depending on what is important to them in life. Measures of wellbeing based on the capability approach are thus based on subjective perception, so that preferences of individual persons have to be determined. It can therefore be assumed that subjective preferences have a major impact on wellbeing.

A number of measures of capability have been published [11–13]. Some of these measures are suitable for generic use [11, 12], while other have been adapted to specific populations [13, 14]. In principle, capability can be measured for all persons, thus the capability approach complements commonly used health-related quality of life measures, such as the EQ-5D [15, 16]. As the capability approach focuses on what persons are able to do and be [8–10], capability measures can be used to represent the quality of lifetime experienced at the end of life [17]. Therefore, the ICECAP-Supportive Care Measure (ICECAP-SCM) has been developed recently for use in the evaluation of palliative and supportive care interventions [18]. The descriptive system of the ICECAP-SCM was developed using in-depth interviews with those at various points along the trajectory towards end of life. It consists of seven attributes relevant for capability at the end of life: choice (about my life and care), love and affection, physical suffering, emotional suffering, dignity, support, and preparation (making the preparations I want to make). The attributes are described by four ordinal levels “never (1)”, “rarely (2)”, “sometimes (3)” and “most of the time (4)” (levels for the ICECAP-SCM attributes ‘physical suffering’ and ‘emotional suffering’ are coded reverse). Overall, it is possible to describe 16,384 (4^7^) different capability states with “1111111” representing the no capability and “4444444” the full capability at the end of life [18].

The ICECAP-SCM can be used to measure the capability at the end of life of a single person. Furthermore, it is possible to compare the capabilities of different interventions with each other on the basis of tariffs. Thereby, the value of each of the capability states can be evaluated within a representative sample of the general population. To use the ICECAP-SCM in economic evaluation, tariffs are scaled between 0 and 1, representing no capability and full capability, respectively. Tariffs are available for the validated UK version of the ICECAP-SCM [19]. The tariffs were derived using a profile case best–worst-scaling (BWS) as well as a discrete choice experiment (DCE) valuation exercise.

In general, a BWS task comprises a single alternative or profile (e.g., end of life capability state) described by a combination of each attributes at a specific level. Participants are shown several such hypothetical scenarios or sets in which the profiles are varied by the attribute level scales and which are determined based on a statistical design. In DCEs, participants are presented with more than one profile in the same set, and are required to choose the most acceptable. Thus, DCEs enable participants to compare several profiles simultaneously [20] and BWS ask participants to choose the best and worst statement within the same profile [21]. DCE data analyzed under the random utility framework are therefore useful to derive compensatory trade-offs between several alternatives. However, for participants of a DCE, it may be difficult to understand the task [22]. For the valuation of quality of life outcomes, it has been suggested that profile case BWS is a more intuitive method for eliciting health state preferences, as it presents the respondent with an easier-to-understand choice task than traditional DCEs [20, 23]. In the case of the ICECAP-SCM in particular, the BWS has been shown to be feasible for use at end of life among patients and proxies using a think aloud study [24]. The respective advantages and disadvantages of BWS and DCE is an ongoing discussion [25]. When deriving tariffs for the UK version of the ICECAP-SCM, 6020 participants of a representative survey of the UK general population were asked to imagine their end of life [19]. For the BWS, the best and worst statement of a set of seven attributes of the ICECAP-SCM were chosen. For the DCE, participants were given a second set of seven attributes of the ICECAP-SCM, which had to be compared with the previous set.

Recently, a German version of the ICECAP-SCM has been introduced and validated [26]. Yet, tariffs for the German version of the ICECAP-SCM have not yet been made available. However, it might not be appropriate to use the UK tariffs in Germany, due to possible cultural differences between the UK and other countries, which have been seen in studies comparing health-related quality of life tariffs. In a recently published research article comparing tariffs of the EQ-5D across different countries [27], the authors concluded that country-specific tariffs should be used to evaluate treatment effects. Indeed cultural differences in valuing health-related quality of life are well known, because culture defines the requirements and expectations for a meaningful life [28]. Likewise, culture may influence capability at the end of life. Therefore, country-specific tariffs of the ICECAP-SCM are needed. Thus, the aim of the current study was to determine tariffs for the ICECAP-SCM based on representative data for the German general population.

## Methods

### Experimental design

The experimental design of the BWS and DCE was the same as that used for the UK valuation of the ICECAP-SCM, so that results could be compared [19, 29]. There are 16,384 (4^7^) possible ICECAP-SCM capability end of life states. The number of scenarios was reduced without loss of information using a Bayesian D-efficient design, using priors from 100 respondents from the UK, and by minimizing the variance–covariance matrix of the maximum likelihood estimator. The final design consisted of 16 sets to be completed by each respondent with each set accompanied by both a BWS and DCE task. For the BWS task in each scenario or set, participants were asked to choose the most and least acceptable statements out of seven attributes of one ICECAP-SCM end of life state. Different scenarios presented profiles with varying attribute levels on an ordinal four level scale with levels “never (1)”, “rarely (2)”, “sometimes (3)” and “most of the time (4)”. For the DCE, participants compared an additional end of life profile described by attributes at the middling state (level 2 and 3) with the previous profile from the BWS, and were asked to choose their preferred scenario. The design ensured that the first eight scenarios were the same for all participants in the study. The other eight scenarios were drawn from one of five blocks of eight sets that were randomly assigned to respondents. Blocks were built to reduce the number of sets per participant from *n* = 48 to *n* = 16. Confounding between individuals and blocks was prevented by avoiding correlations between different blocking variables and the design attributes. The design was developed in Ngene: further details are available in [19].

### Socio-demographic and end of life parameters

Socio-demographic parameters in the analysis included age, gender, education, employment status, professional qualification and marital status. Furthermore, parameters to capture the specific situation at the end of life were evaluated. Religiosity was measured by a visual analogue scale between 0 and 10, representing extreme non-religiosity and extreme religiosity, respectively. Furthermore, participants were asked if someone close to them had died in the last 2 years, if they themselves were diagnosed with a life-limiting illness, or if they care/had cared for someone with a life-limiting illness.

### Sampling and piloting

The survey was conducted using an online panel by the external market research institute, USUMA GmbH, Berlin, Germany. The sample of the online panel was drawn representatively with regard to age, gender and federal state from the adult German general population. Only participants with a statement of consent were approved for the survey and were asked to fill in the BWS and DCE questionnaire. Participants with a fill-in time of less than 3.5 min (13 s per set) were excluded from the analysis in order to ensure quality of the data.

The questionnaire of the survey was based on the validated German version of the ICECAP-SCM [26] and was pilot tested in order to assess the difficulty and the comprehensibility of the tasks for participants. Pilot testing was conducted with 13 employees of the Department of Health Economics and Health Services Research (University Medical Center Hamburg-Eppendorf, Hamburg, Germany), and a further 50 participants of the online panel were interviewed subsequent to their participation in the survey. Participants of the piloting test were asked to rate questions in terms of difficulty and intimacy. Participants were given the option to deny answers of life-limiting illness, death, and religiosity.

### Statistical analyses

DCEs and BWSs are based on random-utility models [30], which aim to model the choices of individuals among discrete sets of alternatives, assuming that preferences among these alternatives can be described by a utility function [20]. Thus, preferences among alternatives of the ICECAP-SCM questionnaire can be described by a conditional logit model representing the particular relevance of each ICECAP-SCM attribute for capability [19, 31]. First, the BWS and DCE data were analyzed using conditional logit models. Following this, the similarity between BWS and DCE data was evaluated to assess whether it is possible to combine both sources of choice data. Again, conditional logit models were used to analyze combined data. In order to increase representativeness of the results for the German general adult population, the German Census 2011 [32] was used to generate weights that compensate for underrepresentation and overrepresentation of observations by means of age, gender, and education. All conditional logit models used were estimated using Latent Gold 5.1 with Choice and Advanced/Syntax add-on. Data preparation and further analyses were conducted using R 3.5.1.

#### Analysis of BWS and DCE data

BWS data were analyzed using a partial rank-ordered scale-adjusted conditional logit latent class model, where the best statement of each set was selected first, and the worst statement from those remaining. Sign changes were used to reflect the contrary meaning of the best and worst decisions. Scale differences between the best and worst decision were adjusted by including a scaling factor in the model. Attributes of the ICECAP-SCM were included as independent variables to evaluate their influence on capability at the end of life. Respondents with similar response patterns were classified using latent classes. Models with convergence towards a stable solution (identifiable and same model fit across different starting values and seeds), low Bayesian Information Criterion (BIC) and judged to provide meaningful classes, were selected. Covariates, such as socio-demographic characteristics and variables concerning statements for the end of life, were first included in the model independently as single parameters. In a second step, multiple socio-demographic parameters were tested on their influence on capability. Variables were integrated using a forward algorithm (Wald test significance level of 5%).

DCE data were analyzed using scale-adjusted conditional logit latent class models. Again, attributes of the ICECAP-SCM were included as independent variables to determine their influence on capability at the end of life. Models with convergence towards a stable solution and the lowest BIC were selected. Again, socio-demographic characteristics and variables concerning statements for the end of life were included as single parameters first, and then as multiple parameters using a forward algorithm.

#### Analysis of combined data

BWS and DCE data can be combined if both choice data are shown to have the same data generating process. If the number of classes and the class-specific influence of attributes of the ICECAP-SCM on capability is similar, it may be possible to combine the two sources of choice data. Model coefficients of the separate analyses of BWS and DCE data represented the influence of scale levels of the ICECAP-SCM attributes on capability. Therefore, coefficients based on BWS data were compared with coefficients based on DCE data using Pearson correlations and scatterplots. The consistency of results between BWS and DCE data was assumed for a linear relationship between coefficients of BWS and DCE data, which indicated a similar influence of scale levels of attributes on capability.

The combined data was first analyzed using a scale-adjusted conditional logit latent class model with main effects. Two-way interactions used to derive tariffs for the ICECAP-SCM in the UK were not included, because solutions were not robust. Thus, the capability utility function ${U}_{n}$ for a respondent *n* is represented by the following equation:

$$U_{n} = \exp \left( {y_{{{\text{BWS}}}} + y_{{{\text{BWS}} - {\text{DCE}}}} } \right)\left[ {{\text{Const}} + \mathop \sum \limits_{i} {\text{ASC}}_{i} + \mathop \sum \limits_{i} \beta_{i} X_{{{\text{in}}}} } \right]$$

where $\mathrm{Const}$ represents the effects coded intercept for the DCE data and ${\mathrm{ASC}}_{i}$ represents the effect coded intercept of the attribute $i$ of the ICECAP-SCM. ${X}_{i}$ are indicators for attribute $i$ for $k=1,\dots , 7$. Differences in scaling between DCE and BWS data were adjusted by the scaling factor ${y}_{\mathrm{BWS}-\mathrm{DCE}}$. Differences in scaling between best and worst data were captured by sign changes. Furthermore, an additional scaling factor ${y}_{\mathrm{BWS}}$ was used to adjust scale differences between best and worst data. Models with convergence towards a stable solution and the lowest BIC were selected. Again, socio-demographic characteristics and variables concerning statements for the end of life were included as single parameters first, and then as multiple parameters using a forward algorithm.

#### Determination of tariffs

First, for each of the 16,484 (4^7^) states, class-specific values for BWS, DCE and combined data were calculated, based on the results of the conditional logit latent class models. Then, the average values across the latent classes were calculated by taking the weighted mean of class-specific values across classes. Finally, the value for each state was transformed to a scale between 0 and 1 by subtracting the value for the no capability state “1111111” from the respective value, and by dividing this difference with the difference of the value for the full capability state “4444444” and the value for the no capability state “1111111”. Descriptive statistics and the intraclass correlation (ICC) were used to compare tariffs based on BWS, DCE and combined data.

### Ethical statement

According to the ethics committee of the Hamburg Medical Chamber, an ethics approval was not required as only anonymized survey data was used.

## Results

Of the 6249 persons contacted, 4329 (69%) participants completed the questionnaire of the survey. Of those participants who completed the survey, 1159 (19%) had a fill-in time of less than 3.5 min and/or did not complete a statement of consent, and were excluded from the analysis. On average, included participants had a fill-in time of 18 min. As participants were surveyed representative for the population size of German federal states, participants living in already overrepresented federal states were no longer surveyed. Between reaching the representative population size of German federal states and closing the survey for particular federal states, another 101 (2%) participants living in the particular federal states were interviewed, thus they were excluded from analysis. Of the remaining 3069 participants, 73 (1%) had missing data and were also excluded from the analysis. Overall, 2996 (47%) participants were analyzed.

Sample characteristics differed in age and education compared with the German general adult population. Persons younger than 35 years and persons older 55 years were underrepresented. Furthermore, survey respondents were more likely to have a higher education level (i.e., intermediate secondary school, technical college, A-level exam) compared with the German general adult population. Therefore, sample characteristics in age, gender and education were adjusted to data from the German Census 2011 [32] using population-specific weights. Mean age of the weighted sample was 2.5 years younger. Furthermore, in the weighted sample, a higher proportion had received a secondary school examination, and a lower proportion an A-level exam, compared with pre-weighting characteristics (Table 1).

**Table 1:** Socio-demographic and end of life characteristics of the sample (*n* = 2996)

Characteristics	Sample	Weighted sample^a^
Gender *n* (%)		
Male	1436 (47.9)	1471 (49.1)
Female	1554 (51.9)	1519 (50.7)
Diverse	6 (0.2)	6 (0.2)
Age mean (SD)	52.3 (12.1)	49.7 (12.1)
Education *n* (%)		
Not graduated	5 (0.2)	23 (0.8)
Secondary school	443 (14.8)	871 (29.1)
Intermediate secondary school	1237 (41.3)	1426 (47.6)
High school	1307 (43.6)	675 (22.5)
Other education	4 (0.1)	1 (0)
Employment *n* (%)		
Employed	1461 (48.8)	1574 (52.6)
Self-employed	189 (6.3)	138 (4.6)
Housewife/househusband	196 (6.5)	218 (7.3)
Not employed	130 (4.3)	190 (6.3)
Retired	856 (28.6)	721 (24.1)
Apprenticeship	118 (3.9)	109 (3.6)
Other employment	46 (1.6)	46 (1.5)
Professional qualification *n* (%)		
No professional qualification	138 (4.6)	207 (6.9)
In professional qualification for less than 1 year	23 (0.8)	39 (1.3)
In professional qualification for less than 2 years	188 (6.3)	219 (7.3)
In professional qualification for less than 3 years	1451 (48.4)	1782 (59.5)
Technical college	359 (12.0)	305 (10.2)
University of applied science/polytechnic	308 (10.3)	160 (5.3)
University	461 (15.4)	236 (7.9)
Post-doc	27 (0.9)	13 (0.4)
Other professional qualification	41 (1.3)	35 (1.2)
Marital status *n* (%)		
Married/registered civil partner	1551 (51.8)	1455 (48.6)
In relationship	326 (10.9)	358 (11.9)
Single	642 (21.4)	754 (25.2)
Divorced	380 (12.7)	351 (11.7)
Widowed	97 (3.2)	78 (2.6)
Religiosity mean (SD)	3.6 (5.7)	3.52 (5.1)
Diagnosis of life limiting illness *n* (%)		
Yes	105 (3.5)	109 (3.6)
No	2815 (94.0)	2815 (94.0)
No answer	76 (2.5)	72 (2.4)
Caring for someone with life limiting illness *n* (%)		
Yes	115 (3.8)	108 (3.6)
No	2842 (94.9)	2851 (95.2)
No answer	39 (1.3)	37 (1.2)
Had someone close died in the last two years *n* (%)		
Yes	1229 (41.0)	1171 (39.1)
No	1735 (57.9)	1789 (59.7)
No answer	32 (1.1)	37 (1.2)

^a^The sample was adapted to the German general adult population by means of age, gender, and education using data from the German Census 2011 [32]

### BWS

BWS data were analyzed using a model with two latent classes. While the BIC continued to improve for the third and fourth class, the two latent class model has been kept simpler, with three or four latent classes providing similar content and non-meaningful differences beyond the two latent class model. The final BWS model controlled for differences in gender, age, education, professional qualification, employment, diagnosis of a life-limiting illness, and care for a person with a life-limiting illness in the class membership function. Coefficients for scale levels of attributes were similar in class 1 and differed more widely in class 2 (Table 2). For respondents in class 1, differences in coefficients for scale levels were highest for the attributes ‘physical suffering’ and ‘dignity’, whereas for respondents in class 2, differences in coefficients for scale levels were highest for the attribute ‘love and affection’. Thus, for respondents in class 1 ‘physical suffering’ and ‘dignity’ had the highest influence on capability at the end of life, whereas respondents in class 2 considered ‘love and affection’ to have the highest influence.

**Table 2:** Results of scale-adjusted conditional logit model with two latent classes based on best–worst scaling data (*n* = 2996)

Parameter estimates	Class 1 (26.7%)	Class 2 (73.3%)
Intercept***		
Support	0.388	0.239
Physical suffering	0.989	− 0.148
Preparation	0.214	0.442
Love and affection	0.247	− 0.038
Choice	− 0.227	0.274
Emotional suffering	− 0.613	0.052
Dignity	− 0.999	− 0.821
Choice***		
Never (1)	− 0.243	− 1.850
Only a little (2)	− 0.317	− 1.189
Some (3)	0.101	0.605
Most (4)	0.206	1.863
Love and affection***		
Never (1)	− 0.391	− 2.026
Only a little (2)	− 0.264	− 0.713
Some (3)	0.099	1.026
Most (4)	0.224	2.604
Physical suffering***		
Always (1)	0.426	− 2.450
Often (2)	0.631	− 1.702
Sometimes (3)	0.080	0.116
Rarely (4)	− 0.822	2.166
Emotional suffering***		
Always (1)	0.025	− 1.290
Often (2)	− 0.531	− 1.231
Sometimes (3)	− 0.321	0.342
Rarely (4)	− 0.490	1.267
Dignity***		
Never (1)	− 0.399	− 1.796
Only a little (2)	0.108	− 0.543
Some (3)	0.412	1.498
Most (4)	0.869	2.968
Support***		
Never (1)	− 0.530	− 2.367
Only a little (2)	− 0.306	− 0.970
Some (3)	0.202	0.869
Most (4)	0.407	2.095
Preparation***		
Not any (1)	− 0.342	− 1.605
Only few (2)	0.220	− 0.079
Some (3)	0.456	0.795
Most (4)	0.489	1.597
Gender***		
Male	1.225	− 1.225
Female	0.983	− 0.983
Diverse	− 2.209	2.209
Age, years***	− 0.012	0.012
Education***		
No education	1.100	− 1.100
Secondary school	0.099	− 0.099
Intermediate secondary school	− 0.080	0.080
Technical college	− 0.258	0.258
A level exam	− 0.420	0.420
Other education	− 0.441	0.441
Professional qualification*		
No professional qualification	− 0.043	0.043
In professional qualification for less than 1 year	0.016	− 0.016
In professional qualification for less than 2 years	0.246	− 0.246
In professional qualification for less than 3 years	− 0.139	0.139
Technical college	− 0.165	0.165
University of applied science/polytechnic	− 0.026	0.026
University	− 0.123	0.123
Post-doc	0.266	− 0.266
Other professional qualification	− 0.031	0.031
Employment**		
Employed	− 0.042	0.042
Self-employed	− 0.119	0.119
Housewife/househusband	− 0.169	0.169
Not employed	− 0.019	0.011
Retired	0.235	− 0.235
Apprenticeship	− 0.200	0.200
Other employment	0.376	− 0.376
Diagnosis of a life limiting illness***		
Yes	0.297	− 0.297
No	− 0.231	0.231
No answer	− 0.066	0.066
Caring for someone with life limiting illness***		
Yes	0.105	− 0.105
No	− 0.290	0.290
No answer	0.185	0.185
Class membership parameters	− 0.379	0.379
Scaling factor BWS data***	− 1.113	
Goodness of fit		
LL	− 155,322	
Number of parameters	95	
AIC	310,834	
BIC	311,405	
Number of choice sets	95,872	
Number of subjects	2996	

All attributes and class membership covariates are effects coded. Reference levels reported here are calculated as the negative sum of other parameters of the same attribute. Be careful with the coding for ‘physical suffering’ and ‘emotional suffering’ (levels are coded reverse)

*BWS* best–worst-scaling, *LL* log-likelihood, *AIC* Akaike information criterion, *BIC* Bayesian information criterion.

*p ≤ 0.05, **p ≤ 0.01, ***p ≤ 0.001

### DCE

Solutions based on the DCE data were robust for scale-adjusted conditional logit models with two and three latent classes including age, education, and religiosity as covariates. The BIC was lowest for a model with three latent classes. However, the third class was similar in class composition of respondents and provided similar content. For respondents in class 1, differences in coefficients for scale levels were highest for the attribute ‘love and affection’, whereas for respondents in class 2 differences in coefficients for scale levels were highest for the attribute ‘dignity’ (Table 3). Thus, for respondents in class 1 ‘love and affection’ had the highest influence on capability at the end of life, whereas respondents in class 2 considered ‘dignity’ to have the highest influence.

**Table 3:** Results of scale-adjusted conditional logit model with two latent classes based on discrete choice experiment data (*n* = 2996)

Parameter estimates	Class 1 (66.8%)	Class 2 (33.2%)
Intercept		
Set 1	− 0.156	0.156
Set 2	0.045	− 0.045
Choice***		
Never (1)	− 0.732	− 0.919
Only a little (2)	− 0.143	0.088
Some (3)	0.274	0.225
Most (4)	0.601	0.606
Love and affection***		
Never (1)	− 1.330	− 0.236
Only a little (2)	− 0.105	− 0.290
Some (3)	0.412	0.255
Most (4)	1.024	0.271
Physical suffering***		
Always (1)	− 0.756	− 0.349
Often (2)	− 0.254	− 0.229
Sometimes (3)	0.373	0.269
Rarely (4)	0.638	0.309
Emotional suffering***		
Always (1)	− 0.395	− 0.313
Often (2)	− 0.096	− 0.111
Sometimes (3)	0.225	0.121
Rarely (4)	0.265	0.303
Dignity***		
Never (1)	− 0.805	− 2.763
Only a little (2)	− 0.077	− 0.713
Some (3)	0.235	1.033
Most (4)	0.647	2.442
Support***		
Never (1)	− 0.764	− 0.633
Only a little (2)	− 0.087	− 0.270
Some (3)	− 0.417	− 0.107
Most (4)	0.433	1.010
Preparation***		
Not any (1)	− 0.528	− 0.409
Only few (2)	− 0.050	− 0.084
Some (3)	0.140	0.103
Most (4)	0.438	0.390
Age, years***	− 0.196	0.196
Education***		
No education	− 0.876	0.876
Secondary schools	− 0.095	0.095
Intermediate secondary school	0.045	− 0.045
Advanced technical college certificate	− 0.276	0.276
High school	− 0.329	0.329
Other education	1.530	− 1.530
Religiosity***		
0 (not religious)	− 0.386	0.386
1	− 0.429	0.429
2	− 0.302	0.302
3	− 0.207	0.207
4	− 0.440	0.440
5	− 0.304	0.304
6	− 0.163	0.163
7	− 0.394	0.394
8	− 0.239	0.239
9	− 0.377	0.377
10 (very religious)	0.080	− 0.080
No answer	3.161	− 3.161
Class membership parameters***	1.753	1.753
Scaling factor		
DCE data***	− 1.932	
Goodness of fit		
LL	− 28,527	
#Parameters	64	
AIC	57,182	
BIC	57,567	
Number of choice sets	47,936	
Number of subjects	2996	

All attributes and class membership covariates are effects coded. Reference levels reported here are calculated as the negative sum of other parameters of the same attribute. Be careful with the coding for ‘physical suffering’ and ‘emotional suffering’ (levels are coded reverse)

*DCE* discrete choice experiment, *LL* log-likelihood, *AIC* Akaike information criterion, *BIC* Bayesian information criterion

^*^p ≤ 0.05, **p ≤ 0.01, ***p ≤ 0.001

### Combined model

**Fig. 1:**
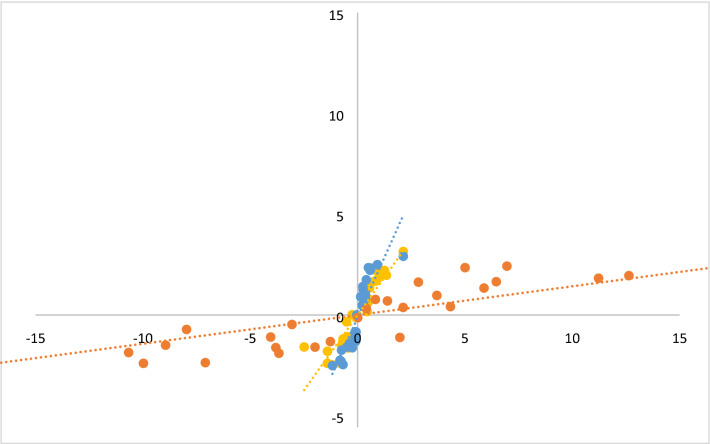
Scatterplot of coefficients for scale levels of the ICECAP-SCM attributes of models based on DCE and BWS data. Comparison of coefficient for scale levels of scale-adjusted conditional logit models with two (yellow), three (blue) and four (orange) latent classes based on DCE (*X*-axis) and BWS (*Y*-axis) data. As coefficients represent the influence of scale levels of attributes on capability, a linear trend (dotted lines) indicate a similar influence of the scale levels for BWS and DCE data on capability

Scatterplots on coefficients for two, three and four latent classes revealed a linear relationship between coefficients based on BWS data and coefficients based on DCE data (Fig. 1). Furthermore, Pearson’s correlation coefficients were 0.91, 0.90 and 0.82 for two, three and four latent classes, respectively.

Combined data was analyzed using a scale-adjusted conditional logit model with two latent classes. The final model for combined data controlled for differences in education, employment, diagnosis of a life-limiting illness, caring for someone with a life-limiting illness and religiosity as covariates (Table 4). For respondents in class 1, differences in coefficients for scale levels were highest for the attributes ‘support’ and ‘dignity’, whereas for respondents in class 2 differences in coefficients for scale levels of attributes were small. Thus, for respondents in class 1 ‘support’ and ‘dignity’ had the highest influence on capability at the end of life, whereas for respondents in class 2, all attributes influenced capability similarly.

**Table 4:** Results of scale-adjusted conditional logit model with two latent classes based on combined best–worst scaling and discrete choice experiment data (*n* = 2996)

Parameter estimates	Class 1 (57.4%)	Class 2 (42.6%)
Intercept***		
Support	− 18.413	− 17.539
Physical suffering	− 18.495	− 17.562
Preparation	− 18.363	− 17.513
Love and affection	− 18.451	− 17.531
Choice	− 18.400	− 17.534
Emotional suffering	− 18.432	− 17.578
Dignity	− 18.449	− 17.546
DCE1	64.423	61.389
DCE2	64.580	61.415
Choice***		
Never (1)	− 0.141	− 0.014
Only a little (2)	− 0.084	− 0.014
Some (3)	0.074	0.015
Most (4)	0.152	0.013
Love and affection***		
Never (1)	− 0.128	− 0.006
Only a little (2)	− 0.048	− 0.003
Some (3)	0.000	− 0.009
Most (4)	0.177	0.018
Physical suffering***		
Always (1)	− 0.114	0.000
Often (2)	− 0.067	− 0.004
Sometimes (3)	0.034	0.005
Rarely (4)	0.146	− 0.001
Emotional suffering***		
Always (1)	− 0.079	0.013
Often (2)	− 0.065	− 0.009
Sometimes (3)	0.049	0.001
Rarely (4)	0.094	− 0.004
Dignity***		
Never (1)	− 0.173	− 0.036
Only a little (2)	− 0.080	− 0.008
Some (3)	0.077	0.019
Most (4)	0.176	0.025
Support***		
Never (1)	− 0.163	− 0.035
Only a little (2)	− 0.058	− 0.017
Some (3)	0.082	0.029
Most (4)	0.140	0.023
Preparation***		
Not any (1)	− 0.146	− 0.017
Only few (2)	− 0.020	− 0.010
Some (3)	0.058	0.006
Most (4)	0.108	0.021
Gender***		
Male	− 0.127	0.127
Female	0.109	− 0.109
Diverse	0.017	− 0.017
Education***		
No education	− 0.988	0.988
Secondary school	0.006	− 0.006
Intermediate secondary school	0.210	− 0.210
Technical college	0.355	− 0.355
A level exam	0.543	− 0.543
Other education	− 0.125	0.125
Employment*		
Employed	0.052	− 0.052
Self-employed	0.221	− 0.221
Housewife/househusband	0.199	− 0.199
Not employed	− 0.032	0.032
Retired	− 0.028	0.028
Apprenticeship	0.005	− 0.005
Other employment	− 0.416	0.416
Diagnosis of a life limiting illness***		
Yes	− 0.338	0.338
No	0.300	− 0.300
No answer	0.038	− 0.038
Caring for someone with life limiting illness**		
Yes	0.107	− 0.107
No	− 0.104	0.104
No answer	− 0.004	0.004
Religiosity*		
1 (extremely non-religious)	− 0.089	0.089
2	0.073	− 0.073
3	0.113	− 0.113
4	− 0.261	0.261
5	− 0.074	0.074
6	− 0.221	0.221
7	0.010	− 0.010
8	− 0.061	0.061
9	− 0.212	0.212
10 (extremely religious)	− 0.355	0.355
No answer	1.051	− 1.051
Class membership parameters	− 0.225	0.225
Scaling factors		
BWS data***	− 2.582	
BWS-DCE data***	1.235	
Goodness of fit		
LL	− 200,256	
Number of parameters	89	
AIC	400,689	
BIC	401,224	
Number of choice sets	143,808	
Number of subjects	2996	

All attributes and class membership covariates are effects coded. Reference levels reported here are calculated as the negative sum of other parameters of the same attribute. Be careful with the coding for ‘physical suffering’ and ‘emotional suffering’ (levels are coded reverse)

*BWS* best–worst-scaling, *DCE* discrete choice experiment, *LL* log-likelihood, *AIC* Akaike information criterion, *BIC* Bayesian information criterion

*p ≤ 0.05, **p ≤ 0.01, ***p ≤ 0.001

### Tariffs

Results of the scale-adjusted conditional logit models based on BWS, DCE and combined data were transformed into tariffs. Tariffs for each capability state can be calculated based on tariff increases and reductions represented in Table 5 or taken from the Appendix Excel worksheet. For example, the tariff for the capability state “1111111” based on BWS data is calculated as follows: The first row of Table 5 provide tariff increases and reductions of 0.008, − 0.004, − 0.003, 0.019, − 0.040, − 0.007 and 0.027 for ‘never being able to make decisions’, ‘never being with people who care about you’, ‘always experiencing physical suffering’, ‘always experiencing emotional suffering’, ‘never experiencing dignity’, ‘never being supported’, and ‘not being prepared’, respectively. Thus, the tariff for the capability state “1111111” based on BWS data is calculated by the sum of the respective tariff increases and reductions: 0.008 – 0.004 – 0.003 + 0.019 – 0.040 – 0.007 + 0.027 = 0. Tariffs for other capability states are derived by adapting the respective tariff increases and reductions. Thus, for example, the tariff for the capability state “1121111” is derived by substituting the tariff reduction of − 0.003 for ‘always experiencing physical suffering’ by the tariff increase of 0.025 for ‘often experiencing physical suffering’. Thus, the tariff for the capability state “1121111” is 0.008 – 0.004 + 0.025 + 0.019 – 0.040 – 0.007 + 0.027 = 0.028 based on BWS data.

**Table 5:** Tariff increases and reductions for scale levels of the ICECAP-SCM attributes to calculate tariffs for ICECAP-SCM capability states based on best–worst-scaling, discrete choice experiment and combined data

Attribute level	Choice	Love and affection	Physical suffering	Emotional suffering	Dignity	Support	Preparation
BWS data							
1	0.008	− 0.004	− 0.003	0.019	− 0.040	− 0.007	0.027
2	0.030	0.043	0.025	0.014	0.010	0.045	0.087
3	0.098	0.108	0.081	0.071	0.085	0.115	0.120
4	0.143	0.164	0.141	0.101	0.142	0.160	0.149
DCE data							
1	− 0.002	− 0.020	0.015	0.041	− 0.069	0.005	0.029
2	0.072	0.061	0.053	0.068	0.049	0.063	0.072
3	0.104	0.115	0.112	0.098	0.129	0.046	0.091
4	0.139	0.157	0.132	0.106	0.204	0.141	0.121
Combined data							
1	0.019	0.016	− 0.024	0.013	− 0.052	− 0.024	0.052
2	0.042	0.052	− 0.009	− 0.006	0.017	0.039	0.111
3	0.139	0.065	0.042	0.051	0.111	0.147	0.161
4	0.169	0.167	0.081	0.064	0.158	0.164	0.198

*BWS* best–worst-scaling, *DCE* discrete choice experiment

The tariffs for the capability state “1112111” based on BWS and combined data were negative, because respondents rated ‘always experiencing emotional suffering’ to be less severe than ‘often experiencing emotional suffering’. As participants never compared both scale levels directly with each other, is was expected that in German the difference between both scale levels may not be pronounced as in English. Therefore, it was assumed that the tariff of the capability state “1112111” is very similar to the tariff of the capability state “1111111”, and was thus it replaced by zero

The tariffs were scaled between 0 and 1, representing the values for the no and full capability states “1111111” and “4444444”, respectively. However, the tariffs for the capability state “1112111” based on BWS data and combined data were negative, because respondents rated ‘always experiencing emotional suffering’ with smaller tariff increases of 0.019 and 0.013 than ‘often experiencing emotional suffering’ with tariff increases of 0.014 and − 0.006, respectively. As differences in tariff increases for both scale levels of the attribute ‘emotional suffering’ were small, social-cultural and linguistic differences were assumed to be responsible for these discrepancies. Participants never compared both scale levels of the attribute ‘emotional suffering’ directly with each other. Furthermore, in German the difference between ‘always experiencing emotional suffering’ and ‘often experiencing emotional suffering’ may not be pronounced as in English. Therefore, it was assumed that the tariff of the capability state “1112111” was very similar to the tariff of the capability state “1111111”, and was thus replaced by zero.

Based on BWS, DCE and combined data, tariffs of all capability states were normally distributed with means and standard derivations (SD) of 0.48 (SD 0.15), 0.53 (SD 0.15) and 0.49 (SD 0.16), respectively. Furthermore, a significant intra-class correlation (ICC) between the tariffs of 0.894 was observed.

## Discussion

This article presents tariffs for the German version of the ICECAP-SCM in order to enable the use of this capability measure in economic evaluations. As the ICECAP-SCM is the first questionnaire to measure capability of persons at their end of life, it enables the evaluation of interventions and services using domains relevant for persons at the end of life.

The current study provides tariffs based on BWS, DCE and combined data. As methods were similar to those used for the UK valuation, the current study is the second study based on combined BWS and DCE data. Differences between tariffs based on BWS, DCE and combined data were small. All three tariffs were distributed normally with a similar mean. Furthermore, the ICCs between tariffs for BWS, DCE and combined data confirmed a strong correlation. However, the coefficients of the various scale levels of the ICECAP-SCM attribute to calculate tariffs varied, especially for those of the attribute ‘dignity’, which may be explained by the different focuses of the BWS and DCE. For BWS, single profiles are compared with each other, whereas sets of profiles are compared by DCEs. Both methods have advantages and disadvantages. Thereby, DCEs are more complex than BWSs by comparing sets of profiles with each other. Therefore, DCEs are able to capture the common influence of attributes. At the same time, DCEs are cognitively more difficult than BWSs and might overburden some participants. Thus, participants may abandon the survey, or answers may depend on the participants’ cognitive ability to understand the DCE tasks. Persons with low education or in extraordinary situations (such as the end of life) might be disadvantaged. In contrast to DCEs, BWSs focus on the best and worst statement in one particular capability state. As BWSs are structured more simply, they are much easier to understand and therefore might lead to more valid results.

The experimental design used in this study was based on the UK valuation exercise. This design was more complex compared with most of the published DCE models [33,33,34]. It allowed two-way interactions for combined data between different attributes of the ICECAP-SCM. Unfortunately, only solutions of the models including interactions between intercepts of the attributes of the ICECAP-SCM and socio-demographic variables were robust. Solutions including interactions between attributes were not robust. The UK study was able to estimate a selected number of interactions for combined data. As the sample size of the UK study (*n* = 6020) was twice as large as the sample size of the current study (*n* = 2996), non-robust solutions in the current study may be due to the smaller sample size. Unfortunately, existing methodological approaches to calculate the required sample size were not transferable to the complex experimental design of the current study. Therefore, robust solutions based on combined data for the current study were derived by analyzing a scale-adjusted conditional logit model with only main effects.

As the statistical analyses based on BWS data was similar for tariffs form German and UK, results are comparable. The tariffs from the UK based on BWS data were normally distributed with mean 0.51, similar to the German tariffs of the current study [19]. Furthermore, the ICC was significant with 0.943, indicating a strong correlation. Yet, coefficients of different scale levels of attributes varied between the German tariffs and the tariffs from the UK based on BWS data. In particular, tariff increases and reductions for different scale levels of the attributes ‘choice’, ‘emotional suffering’, ‘dignity’, and ‘preparation’ varied more for German tariffs than UK tariffs. Thus, German respondents compared with UK respondents graded the influence of ‘choice’, ‘emotional suffering’, ‘dignity’, and ‘preparation’ to be more relevant for capability. On the other hand, tariff increases and reductions for different scale levels of the attributes ‘love and affection’, ‘physical suffering’, and ‘support’ varied less for German tariffs than UK tariffs. Thus, German respondents, when compared with UK respondents, graded the influence of ‘love and affection’, ‘physical suffering’, and ‘support’ to be less relevant for capability. Another difference between the analyses of German and UK data was that fewer latent classes were included into the current analyses, compared with the UK evaluation. Models with four latent classes were chosen for BWS and combined data to determine UK tariffs. Thereby, the UK respondents in class 1 graded the influence of ‘support’ and ‘emotional suffering’ as less relevant for capability based on BWS data. UK respondents in class 2 considered ‘dignity’, ‘choice’ and ‘support’ as most relevant for capability. For UK respondents in class 3 ‘love and affection’, ‘support’ and ‘dignity’ were relevant, whereas UK respondents in class 4 put emphasis on ‘support’ and ‘physical suffering’. Based on combined data, UK respondents in class 1 graded the influenced of ‘support’ as less relevant. UK respondents in class 2 considered ‘love and affection’ and ‘support’ as most relevant for capability. For UK respondents in class 3 ‘physical suffering’ was most relevant, whereas UK respondents in class 4 put emphasis on ‘dignity’ and ‘choice’. German tariffs were based on models with two latent classes. Based on BWS data, German respondents in class 2 graded differences in scale levels of attributes of the ICECAP-SCM higher than German respondents in class 1. Compared with this, German respondents in the DCE graded differences in scale levels of attributes similar in both class.

In conclusion, tariffs for Germany based on BWS, DCE and combined data were similar. However, some differences were observed and have been discussed above. As the analysis of combined data was based on the largest dataset and therefore included the highest amount of information, we would recommend to use tariffs based on combined data in German evaluations. However, as models based on combined data neglected interactions, tariffs based on BWS data may be more comparable between Germany and the UK. This was also suggested for the use of UK tariffs, as former studies for other capability measures (e.g. ICECAP-A [11]) determined tariffs based on BWS data [19].

### Implications

By providing tariffs for the ICECAP-SCM a capability measure is now available to be used as effect measure in health economic analyses in Germany in addition to health-related quality of life. As health-related quality of life measured by the EQ-5D is commonly used to assess health effects in economic evaluations, economic evaluations for end of life interventions should not solely use the ICECAP-SCM. In fact, the use of ICECAP-SCM may compensate for disadvantages of the EQ-5D [7], so that effects of interventions could be captured in a more holistic way. Especially for persons at the end of life the concept of health-related quality of life becomes less relevant, whereas wellbeing becomes more relevant [4–6]. Thus, the capability approach focusing on what persons are able to do and be, depending on what is important to them [8–10], seems to be more suitable to capture effects for interventions at the end of life.

### Strength and limitations

By providing tariffs for the ICECAP-SCM derived from the German general adult population, the ICECAP-SCM will be increasingly practicable for German studies, because tariffs allow to compare the values for capability states at the end of life across medical indications and across countries. Furthermore, the analyses of the current study benefited from a complex experimental design combining DCE and BWS data. Such a complex experimental design allows to capture the best aspects of both approaches: the relative ease of participants to intuitively understand BWS tasks, and the complexity of DCE data, where sets of profiles are compared with each other.

However, as such a complex experimental design has high methodological requirements, the current study has some limitations. First, participants who did not complete the survey or were excluded from the analysis were on average younger and older compared with the German general population. Younger persons (< 35 years old), who did not complete the survey or were excluded from the analyses, may have found it difficult to put themselves in the position of a person at his or her end of life. For older people (> 65 years old) it was expected that these persons were underrepresented in the current study due to the lower affinity with computer technology compared with younger persons in general. Second, the design of the experiments in the current study was based on the design of the UK valuation of the ICECAP-SCM in order to derive comparable results. However, compared with the experimental design of the UK valuation based on combined data, interactions between attributes were not included, thus only main effects in the models with combined data in the current study were included. Models including interactions did not converge and were therefore not included into the current analyses. Even though the experiments have been based on a relatively large sample size of *n* = 2996, a larger sample size might result in robust model solutions. Nevertheless, results of the BWS, DCE and combined data were consistent and a larger sample size was not expected to lead to additional information.

## Conclusions

The capability of persons at the end of life can be evaluated using German tariffs of the ICECAP-SCM. The tariffs allow for comparison of capability at the end of life across medical indications and across countries. Thus, the German ICECAP-SCM can be used as effectiveness measure in health economic analyses in addition to health-related quality of life.

### Supplementary Information

Below is the link to the electronic supplementary material.

Supplementary file1 (XLSX 1343 KB)

## Data Availability

Data cannot be accessed by anyone who is not part of the research team due to ethical and confidentiality concerns.

## Appendix

See Fig. 1.

## Code availability

The code can be provided for the reviewing process if needed.
